# DESIGNING FUTURE HEALTH CARE ENVIRONMENTS FOR AN AGING SOCIETY: TRANSDISCIPLINARY PERSPECTIVES

**DOI:** 10.1093/geroni/igae098.2021

**Published:** 2024-12-31

**Authors:** Felix Kabo, Cameron Contreras

**Affiliations:** Chair; Discussant

## Abstract

As our society grapples with demographic aging, it is imperative that we design healthcare environments that better support the well-being of older adults in the context of critical staffing shortages in the healthcare sector. Beyond aesthetics and visual appeal, thoughtful healthcare design significantly and directly impacts patient outcomes, staff well-being, and the overall quality of care. This symposium brings together four complementary presentations from the design and research fields that employ transdisciplinary perspectives to examine the issue of designing healthcare environments for an aging society. Kabo, Morrison, and Ritsema discuss how digital twins, virtual representations of real-world objects, can be used to transform the future of healthcare design, thus delivering quality care experiences that sufficiently support the needs of older adults. Calkins provides an overview of evaluative research on healthcare design outcomes by focusing on the post-occupancy evaluation process by the SAGE Federation and links the research to the holistic design of age-friendly healthcare environments. Perreault and Kaminski use two innovative case studies to discuss and illustrate best practices in healthcare design for older adults, and how the built environment can be designed as an active aid for patient recovery. Kim will discuss how smart technologies and machine learning can facilitate successful aging-in-place and the design of home environments that better support healthcare for older adults. Collectively, this symposium brings together speakers from different fields (design and research) and disciplines (e.g., gerontology, architecture, interior design, engineering, etc.) to discuss the convergence of design and healthcare for our aging society.

